# Acetaldehyde Induces Cytotoxicity of SH-SY5Y Cells via Inhibition of Akt Activation and Induction of Oxidative Stress

**DOI:** 10.1155/2016/4512309

**Published:** 2015-11-16

**Authors:** Tingting Yan, Yan Zhao, Xia Zhang

**Affiliations:** Department of Bioengineering, Harbin Institute of Technology, Weihai, Shandong 264209, China

## Abstract

Excessive alcohol consumption can lead to brain tissue damage and cognitive dysfunction. It has been shown that heavy drinking is associated with an earlier onset of neurodegenerative diseases such as Alzheimer's disease. Acetaldehyde, the most toxic metabolite of ethanol, is speculated to mediate the brain tissue damage and cognitive dysfunction induced by the chronic excessive consumption of alcohol. However, the exact mechanisms by which acetaldehyde induces neurotoxicity are not totally understood. In this study, we investigated the cytotoxic effects of acetaldehyde in SH-SY5Y cells and found that acetaldehyde induced apoptosis of SH-SY5Y cells by downregulating the expression of antiapoptotic *Bcl-2* and *Bcl-xL* and upregulating the expression of proapoptotic *Bax*. Acetaldehyde treatment led to a significant decrease in the levels of activated Akt and cyclic AMP-responsive element binding protein (CREB). In addition, acetaldehyde induced the activation of p38 mitogen-activated protein kinase (MAPK) while inhibiting the activation of extracellular signal-regulated kinases (ERKs, p44/p42MAPK). Meanwhile, acetaldehyde treatment caused an increase in the production of reactive oxygen species and elevated the oxidative stress in SH-SY5Y cells. Therefore, acetaldehyde induces cytotoxicity of SH-SY5Y cells via promotion of apoptotic signaling, inhibition of cell survival pathway, and induction of oxidative stress.

## 1. Introduction

Excessive alcohol consumption can cause brain tissue damage and cognitive dysfunction [1]. It has been reported that heavy drinking is associated with an earlier onset of neurodegenerative diseases such as Alzheimer's disease (AD) [2]. Acetaldehyde, the most toxic metabolite of ethanol, is speculated to mediate the brain tissue damage and cognitive dysfunction induced by the chronic excessive consumption of alcohol. The major enzyme that generates acetaldehyde during ethanol metabolism in liver is alcohol dehydrogenase. Catalase and cytochrome p450 2E1 are additional enzymes that are important for the local formation of acetaldehyde in the brain [3]. Acetaldehyde can also be directly ingested from foods such as alcohol beverages, fruit juice, and yogurt or from tobacco smoke [4]. The principle enzyme responsible for the detoxification and metabolism of acetaldehyde into acetate is aldehyde dehydrogenase (ALDH) 2. Thus, the local accumulation of acetaldehyde depends on the amount of exposure and the rates of its formation and clearance. Indeed, it has been found that the levels of acetaldehyde in blood are much higher in individuals with defective ALDH2 in comparison with normal individuals after alcohol ingestion [5].

The generation and accumulation of acetaldehyde by local metabolism in brain may contribute to the synaptic dysfunction. It has been demonstrated that acetaldehyde mediates the acute inhibition of long-term potentiation by ethanol in the CA1 region of rat hippocampal slices [6]. The tissue damage caused by acetaldehyde is largely mediated by the direct cytotoxicity caused by acetaldehyde. In rat embryos, acetaldehyde treatment induces marked cell death in several tissues including neuroepithelium, correlating to the malformations seen in fetal alcohol syndrome (FAS) [7]. Similarly, exposure to acetaldehyde inhibits cell growth in primary cultures of rat astrocytes, presumably via apoptotic pathway [8]. Induction of mitochondria dysfunction and overproduction of reactive oxygen species (ROS) have been linked to the action of acetaldehyde [9, 10]. The redox imbalance induced by acetaldehyde is accompanied by a transient reduction in the protein content of mitochondrial superoxide dismutase SOD2 [11]. In rat cerebellar neuron cultures, acetaldehyde treatment causes decrease in cell viability while impairing mitochondrial function and significantly elevating markers of oxidative stress including 4-hydroxy-2-nonenal and 8-hydroxydeoxyguanosine [12]. In addition, it has been shown that acetaldehyde induces cytotoxic effects of neuronal cells by activating apoptotic signals such as cytochrome c release and caspase 3 activation [13].

Above evidence suggests acetaldehyde may cause neurotoxic effects by promoting oxidative stress and apoptotic signals. However, the exact molecular mechanisms of acetaldehyde-induced neurotoxicity are not totally understood. There are few studies on whether acetaldehyde affects pathways that are essential for cell survival such as the activation of Akt (a serine/threonine kinase or protein kinase, PKB) and cyclic AMP-responsive element binding protein (CREB). In this study, we investigated the cytotoxic effects of acetaldehyde in SH-SY5Y cells and found that acetaldehyde induced apoptosis of SH-SY5Y cells by downregulating the expression of antiapoptotic* Bcl-2* and* Bcl-xL* and upregulating the expression of proapoptotic* Bax*. Acetaldehyde treatment led to a significant decrease in the levels of activated Akt and CREB. In addition, acetaldehyde induced the activation of p38 mitogen-activated protein kinase (MAPK) while inhibiting the activation of extracellular signal-regulated kinases (ERKs, p44/p42MAPK). Meanwhile, acetaldehyde treatment led to elevated production of ROS and oxidative stress in SH-SY5Y cells. Therefore, acetaldehyde induces cytotoxicity of SH-SY5Y cells via promotion of apoptotic signaling, inhibition of cell survival pathway, and induction of oxidative stress.

## 2. Materials and Methods

### 2.1. Materials

Fetal bovine serum (FBS), streptomycin, and penicillin were purchased from Thermo Scientific (Rockford, IL, USA). Dulbecco's modified Eagle's medium (DMEM), trypsin, and reverse-transcription reaction system were purchased from Invitrogen (Eugene, OR, USA). Caspase 3 activity kit, BCA protein assay kit, DMSO, BeyoECL plus Western blotting detection system, anti-Akt, and goat antirabbit IgG (H+L) antibodies were purchased from Beyotime Institute of Biotechnology (Haimen, China). MDA assay kit and GSH assay kit were purchased from Nanjing Jiancheng Bioengineering Institute (Nanjing, China). Brandford protein assay kit, anti-phospho-Akt (Ser 473) antibody, agarose, primers, and PCR reaction system were purchased from Sangon Biotech (Shanghai, China). 2′, 7′-Dichlorofluorescin diacetate (DCFH-DA) was purchased from Sigma Chemical (St. Louis, MO, USA). Antibodies for CREB, phospho-CREB (Ser 133), p38MAPK, phospho-p38MAPK (Thr180/Tyr182), JNK, phospho-JNK (Thr183/Tyr185), p44/p42 MAPK, and phospho-p44/p42MAPK (Thr202/Tyr204) were purchased from Cell Signaling Technology (Beverly, MA, USA).

### 2.2. Cell Viability Assay

Human neuroblastoma SH-SY5Y cells were maintained in DMEM with 10% FBS and 1% streptomycin/penicillin in a CO_2_ incubator at 37°C, with 5% CO_2_ and 95% air. Cells were seeded in 12-well plates with a density of 4 × 10^4^ per well. The viability of cells was checked by trypan blue exclusion assay.

### 2.3. Hoechst 33258 Nuclear Staining Assay

Apoptotic cells were detected by Hoechst 33258 nuclear staining assay. Cells were fixed in 4% paraformaldehyde in PBS for 10 min at room temperature after 24 h treatment of acetaldehyde. The fixed cells were incubated with Hoechst 33258 for 5 min at room temperature and subsequently washed with PBS for three times. The fluorescence was examined using an Olympus BX53 fluorescence microscope.

### 2.4. Caspase 3 Activity

The activity of caspase 3 was determined using a caspase 3 activity kit according to the manufacturer's instructions. After treatments, cells were harvested by digesting with trypsin and the cell lysates were prepared. To evaluate the activity of caspase 3, cell lysates were incubated with Ac-DEVD-pNA and reaction buffer for 10 h at 37°C. Substrate cleavage was then measured using a spectrometer at 405 nm.

### 2.5. Measurement of Intracellular Oxidation Stress

Fluorescent probe DCFH-DA was used to determine the intracellular generation of ROS. After 1 h treatment with 10 mM acetaldehyde, cells were rinsed three times with PBS and incubated with 5 *μ*M DCFH-DA at 37°C for 30 min. The fluorescence was examined using an Olympus BX53 fluorescence microscope. The concentrations of malondialdehyde (MDA) and glutathione (GSH) were determined using commercial available kits according to the manufacturer's instructions.

### 2.6. RT-PCR

Total RNA was isolated using RNAiso Plus reagent (Takara Biotechnology (Dalian) Co., Ltd.) and converted to cDNA using reverse-transcription reaction system. PCR was then performed to determine the expression of* Bcl-2*,* Bcl-xL*, and* Bax*. The housekeeping gene* GAPDH* was used as the internal control. The sequences of forward and reverse primers used were ACC ACA GTC CAT GCC ATC AC and ACC TTG CCC ACA GCC TTG for* GAPDH*; CCA GCT GCC TTG GAC TGT GT and GGT TTA TTA CCC CCT CAA GAC CAC for* Bax*; GGA GGA TTG TGG CCT TCT TG and GG TGC CGG TTC AGG TAC TCA for* Bcl-2*; and TTG GAC AAT GGA CTG GTT A and GTA TAG TGG ATG GTC AGT G for* Bcl-xL*. The PCR conditions were 95°C for 5 min followed by 35 cycles at 95°C for 1 min, 55°C for 1 min, and 72°C for 1 min followed by final extension at 72°C for 10 min for* GAPDH*; 94°C for 3 min followed by 25 cycles at 94°C for 30 s, 54°C for 30 s, and 72°C for 1 min followed by final extension at 72°C for 5 min for* Bcl-2*; 95°C for 5 min followed by 40 cycles at 95°C for 1 min, 55°C for 1 min, and 72°C for 1 min followed by final extension at 72°C for 10 min for* Bcl-xL*; and 94°C for 5 min followed by 30 cycles at 94°C for 30 s, 54°C for 30 s, and 72°C for 1 min followed by final extension at 72°C for 5 min for* Bax*. The PCR products were electrophoresed on 1.5% agarose gel, stained with ethidium bromide, and photographed under ultraviolet light.

### 2.7. Western Blot Analysis

After treatments, cells were washed twice with PBS and the cell lysates were prepared in cell lysis buffer (Tris 20 mM, NaCl 150 mM, EDTA 1 mM, sodium pyrophosphate 2.5 mM, NaF 20 mM, *β*-glycerophosphoric acid 1 mM, and sodium orthovanadate 1 mM). The supernatants were collected after centrifugation at 14000 ×g for 10 min at 4°C and the protein concentration of the supernatants was measured. 20 *μ*g of protein extracts was resolved by SDS-polyacrylamide gel electrophoresis and then transferred to polyvinylidene difluoride (PVDF) membrane and subsequently incubated with specific primary antibodies. PVDF membrane was washed by Tris buffered saline (TBS) containing 0.1% Tween-20 for three times. For detection, the PVDF membrane was incubated with a horseradish peroxidase-coupled secondary antibody, followed by an enhanced chemiluminescence substrate reaction using BeyoECL plus Western blotting detection system.

### 2.8. Statistical Analysis

All the experiments were carried out in triplicates. Quantitative data are represented as the mean ± SD. Student's *t*-test was used to compare the difference between the control and treatment groups. *P* values of 0.05 were considered statistically significant.

## 3. Results

### 3.1. Acetaldehyde Induces Apoptosis and Affects the Gene Expression of Bcl-2 Family Proteins

**Figure 1:**
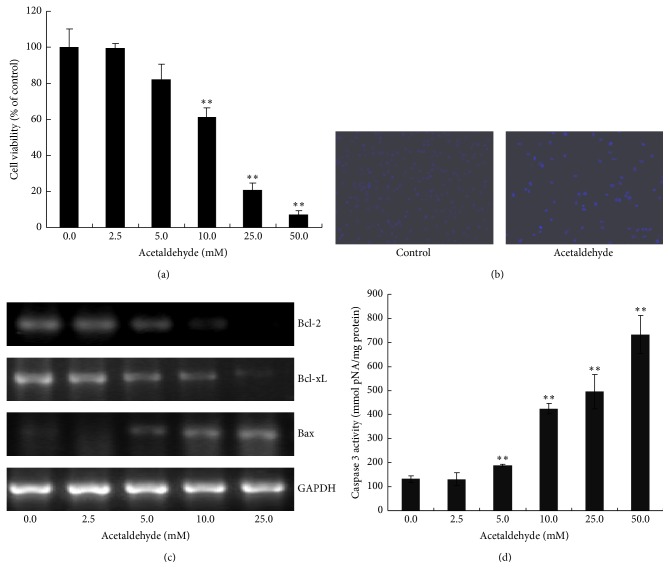
Effects of acetaldehyde on the cell viability and apoptosis of SH-SY5Y cells. Cells were incubated with various concentrations of acetaldehyde for 24 h. (a) Cell viability was determined with trypan blue assay. The number of cells in control group is set to 100%. (b) The cells were fixed and stained with Hoechst 33258. (c) SH-SY5Y cells were treated with different concentration of acetaldehyde for 24 h. Total RNA was isolated after the treatment and the expression of* Bcl-2*,* Bcl-xL*, and* Bax* genes was assessed by RT-PCR. The expression of GAPDH was used as an internal control. (d) Total lysates of cells were collected and the caspase 3 activities were determined. Data are expressed as the values of concentration of pNA. Values are means ± SD, *n* = 3. *∗∗*, significantly different from untreated cells (*P* < 0.01).

We first examined the effect of acetaldehyde on the cell viability of SH-SY5Y cells. As shown in Figure 1(a), acetaldehyde decreased the viability of SH-SY5Y cells significantly and in a concentration-dependent manner, suggesting that acetaldehyde induced cytotoxicity of SH-SY5Y cells. Hoechst 33528 staining of cells treated with 10 mM of acetaldehyde for 24 h demonstrated that acetaldehyde induced apoptosis of SH-SY5Y cells (Figure 1(b)).

Proapoptotic protein Bax and antiapoptotic proteins, Bcl-2 and Bcl-xL, are Bcl-2 family proteins that regulate apoptotic pathway via affecting the permeability of the mitochondrial outer membrane [14]. We next examined the effect of acetaldehyde on the expression of these Bcl-2 family proteins. As shown in Figure 1(c), exposure of SH-SY5Y cells to acetaldehyde for 24 h resulted in the decrease in* Bcl-2* and* Bcl-xL* mRNA levels, which were concomitant with the increase in the level of* Bax*. Thus, acetaldehyde treatment might promote apoptosis by decreasing the expression of antiapoptotic proteins* Bcl-2* and* Bcl-xL* and inducing the expression of proapoptic protein* Bax*. Caspase 3 is a hallmark of the late apoptotic events. As shown in Figure 1(d), acetaldehyde treatment significantly increased the activity of caspase 3 and the elevation of caspase 3 activities by acetaldehyde had a dose-response effect. These data indicated that acetaldehyde treatment induced cytotoxicity and apoptosis of SH-SY5Y cells.

### 3.2. Effect of Acetaldehyde on the Activation of Akt and CREB Pathway

**Figure 2:**
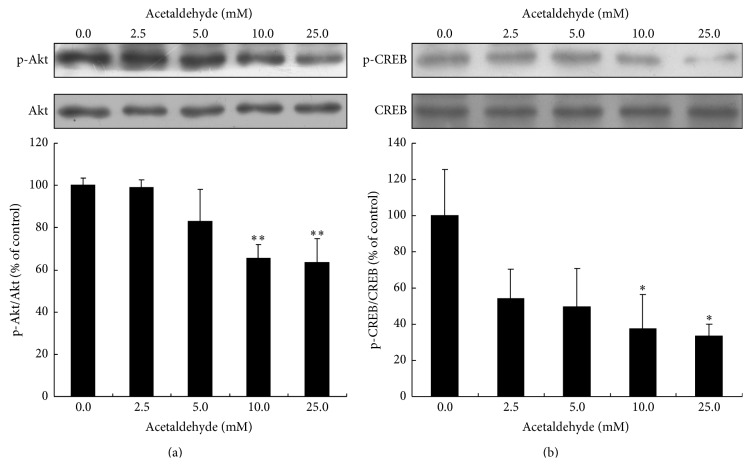
Effects of acetaldehyde treatment on the activation of Akt and CREB in SH-SY5Y cells. SH-SY5Y cells were treated with different concentrations of acetaldehyde for 24 h. Total cell lysates were collected and the amount of Akt, phospho-Akt (Ser473) (a), CREB, and phospho-CREB (Ser 133) (b) was determined by Western blot analysis. The intensities of the bands were quantified by densitometric analyses and normalized by the amount of Akt or CREB. Values are means ± SD from three independent experiments. *∗*, significantly different from control (*P* < 0.05); *∗∗*, significantly different from control (*P* < 0.01).

Akt/CREB pathway is important for the survival of neuronal cells [15]. To find out whether acetaldehyde affects cell survival pathway, the activation of Akt and CREB was examined by Western blot analysis. As shown in Figure 2(a), treatment of acetaldehyde (10 and 25 mM) for 24 h induced a significant decrease in the levels of activated Akt. Similarly, levels of activated CREB were also decreased by the treatment of acetaldehyde (Figure 2(b)). These data suggested that acetaldehyde may decrease cell viability and promote apoptosis by inhibiting the activation of Akt and CREB.

### 3.3. Effect of Acetaldehyde on the Activation of MAPKs

**Figure 3:**
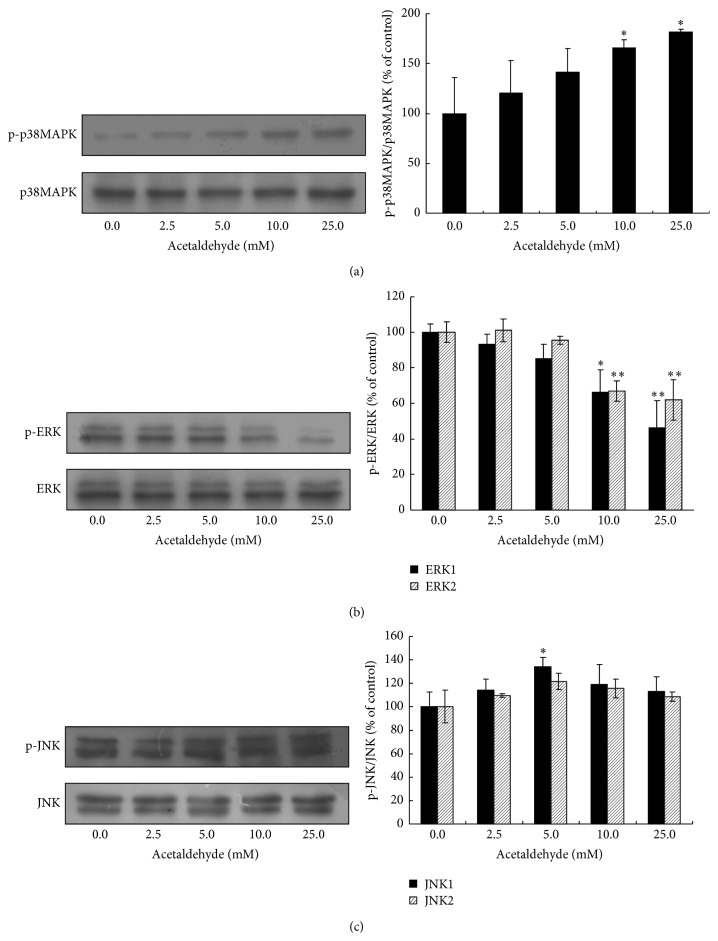
Effects of acetaldehyde on the activation of p38MAPK/ERK/JNK in SH-SY5Y cells. SH-SY5Y cells were treated with different concentrations of acetaldehyde for 24 h. Total cell lysates were collected and the protein levels of p38MAPK, phosho-p38MAPK (Thr180/Tyr182) (a), ERK (p44/p42) MAPK and phospho-ERK (Thr202/Tyr204) (b), and JNK and phospho-JNK (Thr183/Tyr185) (c) were determined by Western blot analyses. The intensities of the bands were quantified by densitometric analyses and normalized by the amount of p38MAPK, JNK, or ERK. Values are means ± SD from three independent experiments. *∗*, significantly different from control (*P* < 0.05); *∗∗*, significantly different from control (*P* < 0.01).

MAPKs have been shown to have important roles in promotion or inhibition of apoptosis. We next examined the effect of acetaldehyde on the activation of p38MAPK/ERK/JNK pathway by Western blot analysis. Treatment of acetaldehyde increased the levels of activated p38MAPK in a dose-dependent manner (Figure 3(a)). In contrast, acetaldehyde treatment caused a downregulation of the levels of activated ERK (Figure 3(b)). There was only a slight change in the activation of JNK after 24 h treatment of acetaldehyde (Figure 3(c)).

### 3.4. Acetaldehyde Increases Oxidative Stress in SH-SY5Y Cells

**Figure 4:**
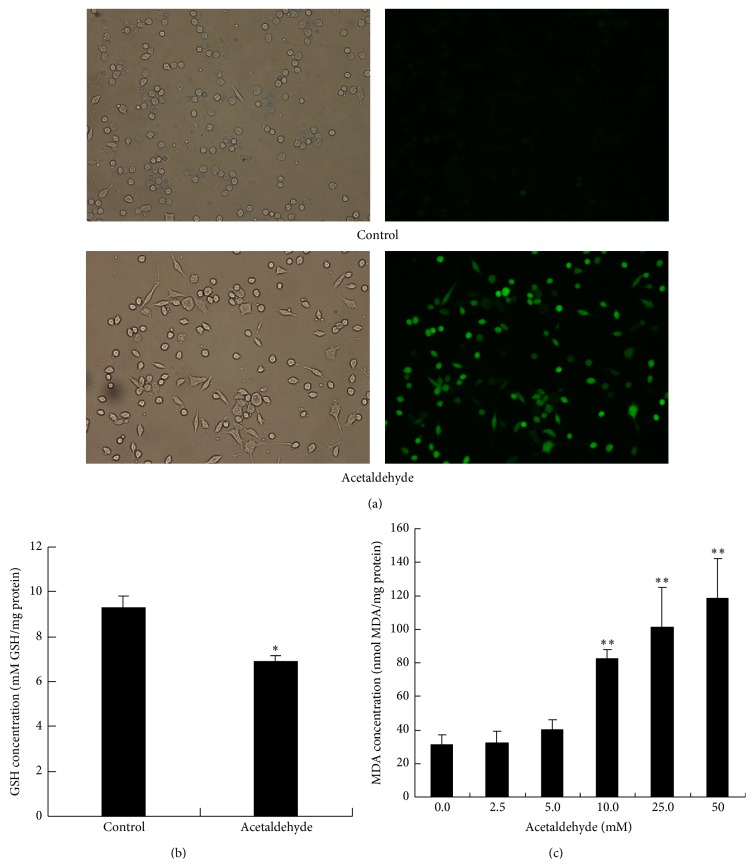
Acetaldehyde increases oxidative stress in SH-SY5Y cells. (a) SH-SY5Y cells were treated with 10 mM of acetaldehyde for 1 h. Cells were then stained with DCFH-DA to determine the production of ROS. Images on the left side are the phase images of the cells. (b) SH-SY5Y cells were treated with 5 mM of acetaldehyde for 2 h. The levels of reduced GSH were determined in the cell lysates. (c) SH-SY5Y cells were treated with different concentration of acetaldehyde for 24 h. MDA production was measured in the cell lysates. Values are means ± SD. *∗*, significantly different from control cells (*P* < 0.05); *∗∗*, significantly different from control cells (*P* < 0.01).

To further study the underlying mechanism of the cytotoxic effects of acetaldehyde, the effects of acetaldehyde on the redox status of the cells were studied. As shown in Figure 4(a), exposure of acetaldehyde caused a quick and dramatic increase in the production of ROS in SH-SY5Y cells. In addition, acetaldehyde treatment also induced a decrease in the concentration of tripeptide GSH (Figure 4(b)), which plays important function in the detoxification of ROS. MDA, which is a breakdown product of the oxidative degradation of cell membrane lipids, is generally considered as the marker of intracellular oxidative stress and an indicator of lipid peroxidation [16]. Thus, we next determined the concentration of MDA in acetaldehyde treated SH-SY5Y cells. As shown in Figure 4(c), MDA levels increased significantly by acetaldehyde treatment in a concentration-dependent manner. The increase of ROS and MDA levels and the decreased GSH concentration suggested that the oxidative stress was induced by the acetaldehyde treatment, which might subsequently lead to the cytotoxicity and apoptosis of SH-SY5Y cells.

## 4. Discussion

Excessive accumulation of acetaldehyde in brain could lead to neurotoxicity and perhaps contribute to the acceleration of the development of neurodegenerative diseases; however, the exact molecular mechanisms of acetaldehyde-induced neurotoxicity are not totally understood. Here we studied the cytotoxic effects of acetaldehyde in SH-SY5Y cells and found that acetaldehyde induced cytotoxicity by promoting apoptosis and inhibiting cell survival pathways.

The role of mitochondria in the apoptotic process has been well established. Mitochondria apoptotic pathway is mainly regulated by Bcl-2 family proteins, including proapoptotic protein Bax and antiapoptotic proteins, Bcl-2 and Bcl-xL, via affecting the permeability of the mitochondrial outer membrane [14]. The balance between antiapoptotic and proapoptotic Bcl-2 family proteins determines the fate of cells including the survival of individual neurons [17–19]. Our results showed that the expression of* Bcl-xL* and* Bcl-2* was decreased while the expression of* Bax* was upregulated by acetaldehyde treatment in SH-SY5Y cells. These results suggested that acetaldehyde induced apoptosis of SH-SY5Y cells though mitochondria apoptotic pathway.

The serine/threonine kinase Akt, also known as protein kinase B (PKB), is a key player in regulating cell signals that are important for cell death and survival [20]. Multiple apoptotic/survival regulating molecules are downstream substrates of Akt, for examples, Bcl-2-associated death protein (Bad) [21], caspase 9 [22], glycogen synthase kinase 3*β* (GSK3*β*) [23], and CREB [24]. The reduction of Akt activation by augmented acetaldehyde exposure has been shown in alcohol-induced myocardial dysfunction and hepatic apoptosis [25–27]. In this study, acetaldehyde treatment caused a dose-dependent inhibition of the activation of Akt in SH-SY5Y cells, suggesting that acetaldehyde might decrease the cell survival and promote apoptotic signaling by suppressing Akt activation. The phosphorylation of transcription factor CREB by Akt on Ser133 results in its transcriptional activation. Our data also showed that the levels of activated CREB were decreased by acetaldehyde treatment. CREB promotes cell survival via a transcription-dependent mechanism, upregulating the expression of antiapoptotic genes such as* Bcl-2* [28, 29]. Thus, it is possible that the decrease of* Bcl-2* gene expression by acetaldehyde treatment seen in our study resulted from the inhibition of acetaldehyde on Akt/CREB pathway.

MAPKs are important cell signals that are involved in both apoptosis and cell survival. It has been shown that the activation of p38MAPK and c-Jun N-terminal kinases (JNKs) promotes apoptosis [30, 31] while ERKs inhibit apoptosis [32]. There are few studies on how acetaldehyde affects the activation of MAPKs in neuronal cells. In this study, 24 h treatment of SH-SY5Y cells with acetaldehyde activated p38MAPK while inhibiting ERKs in a dose-dependent manner. The suppression of ERKs activation has been associated with the decrease of Bcl-2 expression or the ratio of Bcl-2/Bax that leads to the activation of caspase 3 [33, 34]. It is also reported that ERKs suppress the apoptosis of osteosarcoma cells induced by an acidic polysaccharide via activating Bcl-xL [35]. Thus, the inhibition of acetaldehyde on ERK activation may play a role in its modulation of the expression of Bcl-2 family proteins. As p38MAPK was shown to inhibit CREB activated Bcl-2 expression [36], the activation of p38MAPK by acetaldehyde may also contribute to the downregulation of* Bcl-2* expression, potentially mediated by the inhibitory effect of p38MAPK on CREB activation. Furthermore, previous studies have shown that treating cells with ERKs inhibitor attenuates the activation of Akt [37, 38] while the activation of p38MAPK may be involved in the inactivation of PI3K/Akt signaling pathway [39]. Collectively, the evidence suggests that there may be a crosstalk between Akt-CREB and p38/ERKs pathways, and Akt/CREB may be located in the downstream of p38/ERK signaling pathway in acetaldehyde-induced apoptotic event, with p38MAPK playing a role as proapoptotic factor while ERKs acting as an antiapoptotic factor.

Activated JNKs have been shown to suppress the expression of Bcl-2, leading to the release of cytochrome c and triggering apoptosis [40]. In our study, we only found a slight increase in the activation of JNKs after 24 h exposure of acetaldehyde (5 mM). It is possible that acetaldehyde induces the activation of JNKs within short period after the treatment, as reported in the study of Lee and Shukla [41]. This is currently under investigation and the preliminary results showed that the elevation of activated JNK occurred after a short exposure (<1 h) of acetaldehyde (data not shown). And this short term activation of JNK could contribute to the downregulation of Bcl-2 expression and apoptosis induced by acetaldehyde. Overall, these data suggested that the modulation of MAPKs by acetaldehyde plays important roles in acetaldehyde-induced apoptosis in SH-SY5Y cells.

Oxidative stress has emerged as one of the important factors in neuronal cell death in neurodegenerative diseases such as AD. Accumulating evidence has shown that ethanol and acetaldehyde exposure could lead to elevated production of ROS and oxidative stress [42, 43]. Our results also showed that ROS production was significantly elevated in SH-SY5Y cells after a short time exposure of acetaldehyde. The induction of oxidative stress was also indicated by the decreased intracellular GSH content and the increased levels of MDA after acetaldehyde treatment. These data suggest that oxidative stress is one of the early events induced by acetaldehyde in the cell, and the cytotoxicity of acetaldehyde is at least partly caused by its induction of intracellular oxidative stress.

In summary, our results suggest that a complex crosstalk between signaling pathways, such as MAPKs and Akt/CREB, may act together in acetaldehyde-induced apoptotic event. And acetaldehyde induced cytotoxicity of SH-SY5Y cells via promotion of apoptotic signaling, inhibition of cell survival pathway, and induction of oxidative stress. The study also provides evidence that inhibition of oxidative stress by antioxidants may be beneficial for preventing neuronal damage associated with acetaldehyde-induced cytotoxicity which could be resulting from excessive alcohol consumption.

## Abbreviations

ALDH: Aldehyde dehydrogenase
AD: Alzheimer's disease
CREB: Cyclic AMP-responsive element binding protein
ERKs: Extracellular signal-regulated kinases
JNKs: c-Jun N-terminal kinases
MDA: Malondialdehyde
MAPK: Mitogen-activated protein kinase
ROS: Reactive oxygen species.
